# Reference values for generic instruments used in routine outcome monitoring: the leiden routine outcome monitoring study

**DOI:** 10.1186/1471-244X-12-203

**Published:** 2012-11-21

**Authors:** Yvonne WM Schulte-van Maaren, Ingrid VE Carlier, Frans G Zitman, Albert M van Hemert, Margot WM de Waal, Martijn S van Noorden, Erik J Giltay

**Affiliations:** 1 Department of Psychiatry, Leiden University Medical Center, P.O. Box 9600, Leiden, RC, 2300, The Netherlands; 2 Department of Public Health and Primary Care, Leiden University Medical Center, Leiden, The Netherlands

**Keywords:** Reference values, Routine outcome monitoring, Questionnaires, Mood disorders, Anxiety disorders, Somatoform disorders

## Abstract

**Introduction:**

The Brief Symptom Inventory (BSI), Mood & Anxiety Symptom Questionnaire −30 (MASQ-D30), Short Form Health Survey 36 (SF-36), and Dimensional Assessment of Personality Pathology-Short Form (DAPP-SF) are generic instruments that can be used in Routine Outcome Monitoring (ROM) of patients with common mental disorders. We aimed to generate reference values usually encountered in 'healthy' and ‘psychiatrically ill’ populations to facilitate correct interpretation of ROM results.

**Methods:**

We included the following specific reference populations: 1294 subjects from the general population (ROM reference group) recruited through general practitioners, and 5269 psychiatric outpatients diagnosed with mood, anxiety, or somatoform (MAS) disorders (ROM patient group). The outermost 5% of observations were used to define limits for one-sided reference intervals (95^th^ percentiles for BSI, MASQ-D30 and DAPP-SF, and 5^th^ percentiles for SF-36 subscales). Internal consistency and Receiver Operating Characteristics (ROC) analyses were performed.

**Results:**

Mean age for the ROM reference group was 40.3 years (SD=12.6) and 37.7 years (SD=12.0) for the ROM patient group. The proportion of females was 62.8% and 64.6%, respectively. The mean for cut-off values of healthy individuals was 0.82 for the BSI subscales, 23 for the three MASQ-D30 subscales, 45 for the SF-36 subscales, and 3.1 for the DAPP-SF subscales. Discriminative power of the BSI, MASQ-D30 and SF-36 was good, but it was poor for the DAPP-SF. For all instruments, the internal consistency of the subscales ranged from adequate to excellent.

**Discussion and conclusion:**

Reference values for the clinical interpretation were provided for the BSI, MASQ-D30, SF-36, and DAPP-SF. Clinical information aided by ROM data may represent the best means to appraise the clinical state of psychiatric outpatients.

## Background

Routine Outcome Monitoring (ROM) is a method for the continuous monitoring of patients’ symptomatic and functional status. It provides the clinician with systematic information on type and severity of psychiatric complaints before, during, and after treatment. The web-based ROM assessment battery, which is used in the Leiden ROM Study, comprises both generic and disorder-specific measurement instruments. Generic instruments can be used to assess a broad range of psychopathological symptoms, maladaptive personality traits, and quality of life in any patient irrespective of their psychiatric disorder(s) [1]. In contrast, disorder-specific instruments are administered only to those patients who meet the criteria for a particular disorder.

Responsible clinical decision making (e.g., regarding the effectiveness and possible termination of treatment or referral from primary care to specialized mental health care and vice versa), based on ROM assessment, depends on the correct interpretation of the measures. Correct interpretation is only possible if patients’ ROM data can be compared to reliable reference values (from a reference population).

Reference values [2] are often established in healthy populations [3]. Health, a relative condition lacking a universal definition, should nevertheless be clearly defined, a priori, via inclusion and exclusion criteria [4-6]. In non-realistic ‘supernormal’ (i.e., too healthy) reference groups [7] unreasonable narrow reference intervals can be expected. Horn and colleagues (2001) studied the effect of including physician-determined non-healthy individuals in a reference sample. Physician-defined healthy groups with and without non-healthy individuals were compared. Even in healthy samples, outliers may exist. There are marked effects to be expected of non-healthy individuals in the computation of reference values. As non-healthy individuals likely increase the chance of outliers, the width of reference intervals may increase by about 10% [8]. Thus, if non-healthy individuals are included in the reference group, then some subjects would be categorized as having responded to treatment. This would not have happened if only healthy individuals were included. Outlier removal would be an alternative methodology applied in the generation of reference values. Since extreme values can have a profound effect in establishing reference values, sample sizes of at least 120 (after partitioning in relevant subclasses) are needed to reduce the amount of uncertainty and error [9]. Common reference values are means and standard deviations (SDs), which can help to determine whether an individual or a group scores below or above the average of the ‘healthy’ or the ‘psychiatrically ill’ subjects. Also, percentile scores are often used as reference values. These non-parametric values do not rely on Gaussian data distributions [3,9]. The lower interval, bounded by the 95^**th**^ percentile, commonly serves as the reference group [3]. When both reference and patient group data are available, Receiver Operating Characteristics (ROC) analyses can provide additional cut-offs, reflecting the trade-off between sensitivity (measure of positivity; the proportion of actual positives correctly identified as such) and specificity (measure of negativity; the proportion of negatives which are legitimately ruled out) [10].

Some frequently used generic self-report ROM instruments include the Brief Symptom Inventory (BSI) [11,12], the Mood & Anxiety Symptom Questionnaire −30 (MASQ-D30) [13,14], the Short Form Health Survey 36 (SF-36) [15,16], and the Dimensional Assessment of Personality Pathology - Short Form (DAPP-SF) [17,18]. In this generic set of instruments the DAPP-SF is intended not so much for Axis II diagnoses of psychopathology according to the DSM-IV but for the assessment of (dysfunctional) personality traits.

Previous studies mainly reported means and SDs for the general population for the BSI [11,19] and SF-36 [15,20-22], and for the general population and psychiatric patients for the DAPP-SF [18,23], while for the MASQ-D30 no such reference values have been published. Except for the BSI [11], no clinically relevant cut-off scores between ‘healthy’ and ‘psychiatrically ill’ have been reported. In most of the studies the population-based reference groups were relatively small, ranging from 200 [11] to 719 [19] for the BSI, and between 51 [24] and 478 [18,23] for the DAPP-SF, leading to somewhat imprecise reference values [4,8]. Reference values subcategorized according to gender and age were reported for the SF-36 [21,22,25] but they are not available for the BSI, MASQ-D30 or DAPP-SF.

We aimed to establish reference values, means and SDs, percentile scores, and cut-off points, for a comprehensive set of generic ROM instruments that can be offered to every patient referred for (but not necessarily diagnosed with) mood, anxiety, or somatoform (MAS) disorders. These comprise the vast majority of psychiatric patients, notwithstanding those with addiction disorders. In this set, the severity of general psychopathology, (dysfunctional) personality traits, and subjective mental and physical well-being are covered respectively by the BSI, the MASQ-D30, the DAPP-SF, and the SF-36. We tested an apparently healthy population of 1294 subjects who were recruited through general practitioners, and examined similar data from a ‘psychiatrically ill’ population of 5269 outpatients diagnosed with MAS disorders. A novel aspect of the current study is that we could include samples of sufficient size for both the healthy reference and the well-defined psychiatric outpatient group.

## Methods

### Participants

The group of participants comprised a reference sample from the general population (ROM reference group) and a ROM sample of psychiatric outpatients (ROM patient group), as previously described in detail [26].

The ROM reference group consisted of 1294 participants aged 18 to 65 years (62.8% females; mean age=40.3 years; SD=12.6) from the ‘Leiden Routine Outcome Monitoring Study’. The study design, objectives, and methods have been described elsewhere [26,27]. Participants were randomly selected from registration systems of eight general practitioners (GPs) in the province South-Holland, the Netherlands. In the Netherlands, 99.9% of the general population is registered with a GP [28]. Therefore, non-consulting GP patients are a very good representation of the Dutch general population. The ROM reference group was stratified for gender, age, and urbanization-level (62.3% urban), to make the group demographically comparable to the ROM patient group. Invitations for this study were sent to 4840 persons; 1283 could not be contacted and 67 were not included because of time constraints. Of the remaining 3490 potential participants, 1302 were assessed and 1294 generated complete datasets, resulting in a response rate of 37.1%.

The ROM patient group consisted of 5269 psychiatric outpatients, aged 18 to 65 years (64.6% females; mean age=37.7, SD=12.0). They were diagnosed with and treated for one or more MAS disorders in the Leiden University Medical Center (LUMC) Department of Psychiatry or in the Rivierduinen Psychiatric Institute, the regional provider of specialized mental health care.

### Procedures

Procedures for the web-based ROM program of the LUMC Department of Psychiatry are described elsewhere [27,29]. The participants in the ROM reference group were assessed in a similar way to the ROM patient group. Subjects from the ROM reference group completed the self-report instruments BSI, MASQ-D30, and SF-36, and due to time constraints, a random sample of 50% completed the DAPP-SF [26]. The BSI, MASQ-D30, and SF-36 were completed by all 5269 subjects from the ROM patient group, while 234 (4.6%) did not complete the DAPP-SF, again due to time constraints. To facilitate diagnoses of psychopathology according to the DSM-IV, the proceduret for the two groups included a standardized diagnostic interview (i.e., the Mini-International Neuropsychiatric Interview plus (MINI-Plus 5.0.0.) [30,31]). The Medical Ethical Committee of the LUMC approved the general study protocol regarding ROM, in which ROM was organized as part of the treatment process for patients. It involved a comprehensive protocol (titled “Psychiatric Academic Registration Leiden database”) which safeguarded the anonymity of patients and participants and ensured proper handling of the ROM data. All patients gave permission for the use of their ROM data for scientific purposes (written informed consent for this study was not required). In addition, participants of the ROM reference group (non-patients) signed informed consent for the purpose of this study.

### Instruments

The BSI, a short version of the Symptom Checklist (SCL-90) [19], measures psychopathological symptoms. The BSI consists of 53 items divided into 9 subscales: Somatization (SOM), Obsessive-Compulsive (O-C), Interpersonal Sensitivity (I-S), Depression (DEP), Anxiety (ANX), Hostility (HOS), Phobic Anxiety (PHOB), Paranoid Ideation (PAR), and Psychoticism (PSY). Item scores range from 0 (“not-at-all”) to 4 (“extremely”). The subscale and total scores are calculated as an average of the relevant items, with higher scores indicating more severe psychopathology.

The MASQ-D30 measures the dimensions of Clark and Watson’s tripartite model, covering both shared and distinct symptoms of depression and anxiety [13,14]. The MASQ-D30 consists of 30 items, divided into three subscales: Negative Affect (NA), associated with both depression and anxiety; lack of Positive Affect (PA), associated with depressive moods; and Somatic Arousal (SA), associated with anxiety. The items are rated on a 5-point Likert scale, with scores ranging from 1 (“not at all”) to 5 (“extremely”). Subscale scores are calculated as the sum of the relevant items, ranging from 10 to 50, with higher scores indicating more severe psychopathology.

The SF-36, derived from the Rand Medical Outcome Study (MOS) [15,16], measures functional health status and well-being. It can be used as a population-based assessment of quality of life. The SF-36 consists of 36 items divided into eight subscales: Physical Functioning, Role limitations due to Physical health problems (Role-Physical), Bodily Pain, Social Functioning, General Mental Health (Mental Health), Role limitations due to Emotional problems (Role-Emotional), Vitality, General Health Perceptions (General Health) and a question about perceived change of health during the last year (Health Transition). Subscale scores are calculated as the sum of the relevant items, ranging from 0 to 100, with higher scores indicating better functioning.

The DAPP-SF, the short form of the Dimensional Assessment of Personality Pathology – Basic Questionnaire (DAPP-BQ) [17,18], measures personality pathology. It consists of 136 items divided into 18 subscales: Submissiveness, Cognitive Distortion, Identity Problems, Affective Lability, Stimulus Seeking, Compulsivity, Restricted Expression, Callousness, Oppositionality, Intimacy Problems, Rejection, Anxiousness, Conduct Problems, Suspiciousness, Social Avoidance, Narcissism, Insecure Attachment, and Self-harm. Item scores range between 1 (“very unlike me”) and 5 (“very like me”). Subscale scores are calculated as an average of the relevant items, ranging from 1 to 5, with higher scores indicating more maladaptive personality traits.

The Dutch version of the Mini-International Neuropsychiatric Interview plus (MINIplus 5.0.0.) [30,31] was used to establish the presence of Axis I diagnoses according to the Diagnostic and Statistical Manual of Mental Disorders (DSM-IV). This standardized diagnostic interview comprises 23 modules for mood, anxiety, psychotic, somatoform, and eating disorders.

### Statistical analyses

Means, standard deviations (SDs), and percentile scores were calculated for the two samples separately, while ROC analyses were performed in the combined groups. In both samples, subjects with 1 or more missing values per subscale were excluded. This allowed us to conduct a robust evaluation of the use of the instruments. The occurrence of missing values is not completely random, and it depends on unobserved predictors. Therefore we decided to use an almost complete-case analysis, as bias due to missing values was likely to be small due to the small percentage (i.e., 0.01%) of cases that needed to be excluded.

A descriptive analysis of sociodemographic and psychopathological variables was performed, using percentages in the case of categorical variables and means and SDs for the continuous variables. Internal consistency was assessed using Cronbach’s alpha, with >0.70 indicating adequate internal consistency. ROC analyses provided cut-off scores, indicating an optimal discrimination threshold between ‘healthy’ (reference population) and ‘psychiatrically ill’ (psychiatric outpatients). The cut-off was chosen at the value representing equal sensitivity and specificity, since this is the point that yields the best compromise between specificity and sensitivity, with the lowest number of false results (false positive plus false negative). The areas under the ROC curve (AUCs) were calculated to indicate the discriminatory power of the instrument (sub)scales, where AUCs over 0.75 were considered clinically useful with 0.85 showing moderate discriminatory power and 0.95 very high discriminatory power [32]. Furthermore, means and SDs were calculated, together with 5^th^, 25^th^, 50^th^, 75^th^ and 95^th^ percentile scores. When instruments merely assess the level of dysfunctionality, and the discriminative power to detect the level of ‘health’ or normal functionality is limited (i.e., no persons can be earmarked as ‘abnormally healthy or good functioning’), the lowest 2.5% is irrelevant. Therefore, the top 5% (or lower 5% in case of SF-36 subscales) was chosen as representing ‘abnormal’. Reference values were also presented for 4 subgroups: young women (aged 18–40 years), older women (aged 41–65 years), young men (aged 18–40 years), and older men (aged 41–65 years). SPSS for Windows version 17.0 (SPSS Inc., Chicago, IL, USA) was used for data analysis. To test our decision not to exclude those individuals in the ROM reference group with a current psychiatric diagnosis, we performed a sensitivity analysis.

## Results

### Sociodemographic and psychiatric characteristics of the samples

The sociodemographic and psychiatric characteristics of the ROM reference group and the ROM patient group are shown in Table 1.

**Table 1:** Sociodemographic and psychiatric characteristics of the ROM reference (n=1294) and patient (n=5269) groups

	**ROM reference group**	**ROM patient group**
Gender (%)		
Male	481 (37.2)	1864 (35.4)
Female	813 (62.8)	3405 (64.6)
Age (mean, SD) in years	40.3 (12.6)	37.7 (12.0)
Male	41.3 (12.6)	39.1 (11.9)
Female	39.8 (12.6)	36.9 (12.0)
Marital status (%)		
Married/cohabitating	890 (68.8)	25.19 (47.8)*
Divorced/separated/widow	77 (6.0)	688 (13.1)*
Single	327 (25.2)	1730 (32.8)*
Housing situation (%)		
Living alone	201 (15.5)	1128 (21.4)*
Living with partner	903 (69.8)	2568 (48.7)*
Living with family	190 (14.7)	1241 (23.6)*
Educational status (%)		
Lower	295 (22.8)	2112 (40.1)*
Higher	999 (77.2)	2824 (53.6)*
Employment status (%)		
Employed part-time	512 (39.6)	1141 (21.7)*
Employed full-time	552 (42.7)	1105 (21.0)*
Unemployed/retired	194 (15.0)	1337 (25.4)*
Work-related disability (%)	36 (2.7)	1354 (25.7)*
Ethnic background (%)		
Dutch	1163 (89.9)	4335 (82.3)
Other ethnicity	131 (10.1)	934 (17.7)
MINI diagnoses (%)		
Currently None	1153 (89.1)	0**
Anxiety disorder	53 (4.1)	1449 (27.5)
Mood disorder	7 (0.5)	1573 (29.9)
Somatoform disorder	41 (3.2)	403 (7.6)
Anxiety & Mood disorders	7 (0.5)	1257 (23.9)
Anxiety & Somatoform disorders	9 (0.7)	172 (3.3)
Mood & Somatoform disorders	1 (0.1)	228 (4.3)
Anxiety & Mood & Somatoform	2 (0.2)	187 (3.5)
Total Anxiety disorder	71 (5.5)	3065 (58.2)
Total Mood disorder	17 (1.3)	3245 (61.6)
Total Somatoform disorder	53 (4.1)	990 (18.8)

ROM: Routine outcome monitoring.

*No data from 332 (6.3%) patients.

**Selection criterion.

Mean age (40.3 years versus 37.7 years, p<.001) and gender distribution (62.8% females versus 64.6% females, p=.80) were comparable for the ROM reference group and the ROM patient group, as expected due to the stratification. The ROM reference group showed higher levels of education (77.2% versus 53.6% higher education), were more often married (68.8% versus 47.8%), and were less often living alone (15.5% versus 21.4%) relative to the ROM patient group. Unemployment and work-related disability were less prevalent in the ROM reference group (17.7% versus 51.1%). In keeping with our decision to exclude patients without a MINI diagnosis, all subjects from the ROM patient group had at least one DSM-IV disorder. In the ROM reference group, on the other hand, 10.9% had a DSM-IV disorder.

### Internal consistency

The internal consistencies of the instrument subscales (for all subjects combined) are shown in Table 2. None of the subscales had Cronbach’s alphas below the critical cut-off of 0.70, indicating adequate internal consistency.

**Table 2:** Internal consistency and cut-off scores in combined ROM reference (n=1294) and patient (n=5269) groups for four generic Routine Outcome Monitoring instruments

	**Number of items**	**Cronbach’s Alpha**	**ROC analysis cut-off**	**Area under the Curve AUC**	**Sensitivity / specificity**
**Brief Symptom Inventory (BSI)**					
Somatization (SOM)	7	0.86	0.23	0.87	0.80
Obsessive-Compulsive (O-C)	6	0.88	0.69	0.91	0.84
Interpersonal Sensitivity (I-S)	4	0.83	0.54	0.88	0.81
Depression (DEP)	6	0.91	0.50	0.93	0.87
Anxiety (ANX)	6	0.89	0.50	0.92	0.85
Hostility (HOS)	5	0.86	0.30	0.82	0.75
Phobic Anxiety (PHOB)	5	0.83	0.25	0.90	0.84
Paranoid Ideation (PAR)	5	0.84	0.37	0.83	0.76
Psychoticism (PSY)	5	0.77	0.37	0.92	0.85
BSI total score*	53	0.97	0.48	0.96	0.90
**MASQ-D30**					
General distress (GD)	10	0.84	19.0	0.96	0.90
Anhedonic depression (AD)	10	0.92	23.0	0.88	0.80
Anxious arousal (AA)	10	0.74	18.0	0.99	0.96
Short Form 36 (SF36)*					
Physical Functioning	10	0.92	93.5	0.76	0.68
Role-Physical	4	0.88	82.5	0.82	0.78
Bodily Pain	2	0.87	83.7	0.72	0.68
Social Functioning	2	0.85	72.9	0.92	0.79
Mental Health	5	0.90	63.0	0.95	0.89
Role-Emotional	3	0.83	79.6	0.88	0.88
Vitality	4	0.84	52.5	0.92	0.85
General Health	5	0.84	67.5	0.82	0.76
**DAPP-SF:**					
Submissiveness	8	0.87	2.40	0.76	0.71
Cognitive Distortion	6	0.84	1.55	0.83	0.76
Identity Problems	6	0.87	2.08	0.90	0.83
Affective Lability	8	0.86	2.56	0.85	0.77
Stimulus Seeking	8	0.81	1.94	0.55	0.54
Compulsivity	8	0.84	2.69	0.60	0.57
Restricted Expression	8	0.82	2.75	0.78	0.71
Callousness	10	0.79	1.65	0.53	0.51
Oppositionality	9	0.87	2.22	0.79	0.73
Intimacy Problems	9	0.79	2.18	0.60	0.57
Rejection	8	0.83	2.36	0.56	0.55
Anxiousness	6	0.84	2.64	0.85	0.78
Conduct Problems	7	0.73	1.14	0.57	0.56
Suspiciousness	7	0.90	1.40	0.78	0.72
Social Avoidance	6	0.88	2.20	0.80	0.73
Narcissism	8	0.82	2.20	0.56	0.55
Insecure Attachment	6	0.89	2.10	0.80	0.74
Self-Harm	6	0.89	1.08	0.75	0.57

*Higher score corresponds with better functioning.

ROM: Routine outcome monitoring.

MASQ-D30: Mood & Anxiety Symptom Questionnaire 30-item short adaptation; DAPP-SF : Dimensional Assessment of Personality Pathology – short form;

The optimal cut-off derived by the ROC analysis is defined by equal sensitivity and specificity scores.

*The BSI total score comprises 4 additional items next to the subscale items.

### Reference values

#### Percentiles, means and SDs

Table 3 presents the percentile scores and mean values of the BSI, SF-36 and MASQ-D30 subscales for the ROM reference group and the ROM patient group. For the ROM reference group, the distribution of each total score and subscale score was positively skewed, showing apparent health. This was also demonstrated by the substantial percentage of participants having the lowest possible scores (highest for the SF-36).

**Table 3:** Percentile scores and mean values for generic Routine Outcome Monitoring instruments in the ROM reference (n=1294) and patient (n=5269) groups

	**ROM reference group**						**ROM patient group**					
	**P**_**5**_	**P**_**25**_	**P**_**50**_**(median)**	**P**_**75**_	**P**_**95**_	**Mean ± SD**	**P**_**5**_	**P**_**25**_	**P**_**50**_**(median)**	**P**_**75**_	**P**_**95**_	**Mean ± SD**
**Brief Symptom Inventory (BSI)**			n=1294						n=5269			
Somatization (SOM)	0.00	0.00	0.00	0.29	0.71	0.17 ± 0.28	0.00	0.43	0.86	1.43	2.71	1.03 ± 0.83
Obsessive-Compulsive (O-C)	0.00	0.00	0.17	0.50	1.17	0.35 ± 0.42	0.33	1.00	1.67	2.33	3.33	1.67 ± 0.95
Interpersonal Sensitivity (I-S)	0.00	0.00	0.00	0.50	1.00	0.29 ± 0.42	0.00	0.75	1.50	2.25	3.50	1.56 ± 1.04
Depression (DEP)	0.00	0.00	0.00	0.33	0.83	0.20 ± 0.34	0.17	0.83	1.67	2.50	3.50	1.68 ± 1.01
Anxiety (ANX)	0.00	0.00	0.17	0.33	0.83	0.22 ± 0.34	0.17	0.83	1.33	2.17	3.33	1.49 ± 0.94
Hostility (HOS)	0.00	0.00	0.20	0.20	0.80	0.20 ± 0.29	0.00	0.20	0.80	1.40	2.80	0.94 ± 0.86
Phobic Anxiety (PHOB)	0.00	0.00	0.00	0.20	0.60	0.11 ± 0.23	0.00	0.40	1.00	1.60	3.00	1.15 ± 0.93
Paranoid Ideation (PAR)	0.00	0.00	0.00	0.40	0.80	0.23 ± 0.35	0.00	0.40	1.00	1.80	3.00	1.15 ± 0.94
Psychoticism (PSY)	0.00	0.00	0.00	0.20	0.80	0.14 ± 0.28	0.20	0.60	1.20	1.80	2.80	1.23 ± 0.81
BSI total score	0.00	0.06	0.13	0.28	0.68	0.21 ± 0.25	0.34	0.79	1.23	1.75	2.66	1.33 ± 0.71
**MASQ-D30**			n=1294						n=5269			
General distress (GD)	10	11	12	15	23	13.8 ± 4.4	17	23	28	33	40	28.1 ± 6.9
Anhedonic depression (AD)	10	14	17	22	29	18.4 ± 5.8	17	24	31	37	44	30.7 ± 8.3
Anxious arousal (AA)	10	10	11	13	17	11.9 ± 3.0	18	26	31	37	43	31.3 ± 7.5
Short Form 36 (SF36)*			n=1294						n=5269			
Physical Functioning	65	90	100	100	100	92.6 ± 14.2	25	60	80	95	100	74.8 ± 23.7
Role-Physical	5	100	100	100	100	87.0 ± 27.2	0	0	25	75	100	37.2 ± 39.7
Bodily Pain	54	78	90	100	100	86.4 ± 17.6	20	45	67	90	100	65.9 ± 27.5
Social Functioning	63	88	100	100	100	89.9 ± 15.6	0	25	50	63	88	44.8 ± 26.1
Mental health	56	72	80	88	96	79.7 ± 12.3	12	28	40	52	76	41.5 ± 18.2
Role-Emotional	33	100	100	100	100	90.4 ± 24.8	0	0	0	33	100	28.2 ± 36.2
Vitality	40	60	70	80	90	68.6 ± 15.3	5	20	35	45	65	34.3 ± 17.8
General Health	45	65	80	90	100	76.2 ± 16.3	20	35	50	65	90	51. 6 ± 21.0
**DAPP-SF**			n=635						n=5035			
Submissiveness	1.13	1.50	2.00	2.50	3.50	2.10 ± 0.75	1.25	2.25	3.00	3.63	4.38	2.94 ± 0.94
Cognitive Distortion	1.00	1.00	1.17	1.50	2.33	1.36 ± 0.51	1.00	1.50	2.33	3.00	4.17	2.36 ± 0.96
Identity Problems	1.00	1.00	1.33	1.83	2.70	1.54 ± 0.59	1.33	2.33	3.17	3.83	4.67	3.12 ± 1.02
Affective Lability	1.00	1.38	1.88	2.50	3.50	2.01 ± 0.76	1.63	2.63	3.38	3.88	4.63	3.24 ± 0.88
Stimulus Seeking	1.10	1.38	1.88	2.38	3.38	1.99 ± 0.72	1.00	1.50	2.00	2.63	3.75	2.13 ± 0.81
Compulsivity	1.38	2.00	2.50	3.13	4.00	2.58 ± 0.77	1.38	2.13	2.88	3.63	4.50	2.89 ± 0.94
Restricted Expression	1.25	1.75	2.25	2.88	3.63	2.33 ± 0.75	1.75	2.63	3.25	3.88	4.63	3.23 ± 0.86
Callousness	1.00	1.30	1.60	2.00	2.60	1.69 ± 0.50	1.00	1.30	1.70	2.10	2.90	1.77 ± 0.60
Oppositionality	1.00	1.40	1.80	2.30	3.20	1.91 ± 0.65	1.40	2.20	2.80	3.50	4.30	2.83 ± 0.89
Intimacy Problems	1.13	1.63	2.13	2.50	3.38	2.14 ± 0.67	1.13	1.75	2.38	2.88	4.00	2.42 ± 0.85
Rejection	1.38	1.88	2.50	3.00	3.75	2.47 ± 0.76	1.13	1.63	2.25	2.88	3.75	2.31 ± 0.82
Anxiousness	1.00	1.33	1.83	2.50	3.50	2.03 ± 0.81	1.67	2.67	3.50	4.00	4.83	3.37 ± 0.94
Conduct Problems	1.00	1.00	1.13	1.38	2.13	1.26 ± 0.37	1.00	1.00	1.25	1.63	2.63	1.43 ± 0.57
Suspiciousness	1.00	1.00	1.13	1.50	2.15	1.32 ± 0.46	1.00	1.38	2.00	2.88	4.00	2.18 ± 0.99
Social Avoidance	1.00	1.17	1.67	2.17	3.33	1.82 ± 0.73	1.17	2.17	3.00	3.83	4.67	2.98 ± 1.07
Narcissism	1.00	1.63	2.13	2.63	3.50	2.18 ± 0.76	1.10	1.75	2.25	2.88	3.88	2.36 ± 0.83
Insecure Attachment	1.00	1.17	1.50	2.17	3.33	1.74 ± 0.77	1.00	2.00	2.83	3.83	4.83	2.91 ± 1.13
Self-Harm	1.00	1.00	1.00	1.00	1.50	1.07 ± 0.27	1.00	1.00	1.33	2.33	3.67	1.76 ± 0.96

*Higher score corresponds with better functioning.

ROM: Routine outcome monitoring.

MASQ-D30 denotes Mood & Anxiety Symptom Questionnaire 30-item short adaptation; DAPP-SF denotes Dimensional Assessment of Personality Pathology – short form.

P denotes percentile; SD denotes standard deviation.

To calculate sum scores for the DAPP-SF subscales, multiply the mean scores by the number of items per subscale.

For apparently healthy individuals, the mean of cut-off (P_95_) values was 0.82 for the BSI subscales, 23 for the three MASQ dimensions, 45 for the SF-36 subscales, and 3.1 for the DAPP-SF subscales. By contrast, the mean of P_5_ values for the SF-36 subscales was 45.

The BSI subscale scores ranged between 0 and 4. The P_95_ reference scores for the BSI subscales ranged between 0.60 for Phobic Anxiety (PHOB) and 1.17 for Obsessive-Compulsive (O-C) 1.17; for the BSI total score it was 0.68. For six of the nine subscales, the median value (P_50_) was equal to the minimum possible score of 0.

The MASQ-D30 subscale scores ranged between 10 and 50. The P_95_ reference scores for the three MASQ-D30 subscales were: General Distress (GD) - 23; Anhedonic Depression (AD) - 29; and Anxious Arousal (AA) - 17.

The SF-36 subscale scores ranged between 0 and 100, with higher scores indicating better health. Therefore the P5 indicates the cut-off for a low level of functioning. The P_5_ reference scores for the SF-36 subscales ranged between 65 for Physical functioning and 33 for Emotional problems, with the exception of the P_5_ value for Physical health problems, which was 5. The scales that measure well-being as well as health-related limitations (General Health,Vitality,Mental health) showed lower average values, as expected [33]. The other five health-related disability scales had the highest mean subscale scores. For four of the eight subscales, the median value (P_50_) was equal to the maximum possible score of 100.

The DAPP-SF subscale scores ranged between 1 and 5. The range of P_95_ reference scores for the 18 subscales was between 1.50 for Self-Harm and 4.00 for Compulsivity.

Analyses of gender and age indicated that advancing age was associated with more symptoms of psychopathology for both sexes (see Tables 1 through 4 in the Additional file 1, available with the full text of this article). There was a tendency for healthy women to show higher cut-off scores on the BSI and the MASQ-D30 relative to healthy men, while the two sexes showed a different pattern of cut-off scores on the DAPP-SF. Men, and especially young men, reported better health as reflected in higher scores on several subscales of the SF-36.

In a sensitivity analysis, we excluded all 122 (9.5%) subjects in the ROM reference group who had a MINI-diagnosis. Among the remaining 1161 subjects, we found that the median scores on the BSI total score, MASQ-D30 subscales, SF-36 subscales, and DAPP-SF subscales changed on average 2% (interquartile range 1 to 6%). The median P_95_ scores (P_5_ score for the SF36) changed on average 5% (interquartile range 0 to 18%).

#### Receiver operating characteristic (ROC) curves

The results of the ROC analyses are presented in Table 2.

**Figure 1:**
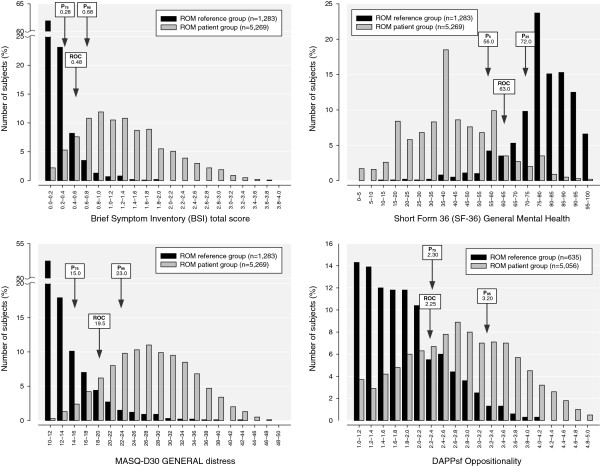
**Distribution of the scores of Brief Symptom Inventory (BSI) total scale, and the subscales of Short Form-36 (SF-36) General Mental Health, Mood and Anxiety Symptom Questionnaire 30 (MASQ-D30) General Distress and Dimensional Assessment of Personality Pathology - Short Form (DAPP-SF) Oppositionality.** Three types of cut-off points are depicted: the 75th percentile score (P_75_), the 95th percentile score (P_95_) and the Receiver Operating Characteristics (ROC) cut-off point defined by equal sensitivity and specificity. Note: in the SF-36 a higher score corresponds with better functioning.

BSI*:* The cut-off point of the BSI total score, which discriminated the ROM reference group from the ROM patient group, was 0.48, with a sensitivity and specificity of 90%. Therefore, for subjects without psychopathology, 10% with a total score of 0.48 or higher would be classified wrongly as a patient with psychopathology. By the same token, the 10% of subjects from the ROM patient group with a total score of 0.48 or lower would be classified wrongly as a psychiatrically ‘healthy’ subject. The AUC values showed that all BSI subscales performed well in making a distinction between patients and non-patients. The discriminating performance of the total score was excellent (AUC=0.96). The best performing subscale was DEP, followed by ANX and PSY. The HOS and PAR subscales showed the least distinctiveness but might perform better in specific subpopulations of patients. Figure 1 presents the discriminative power of the BSI total score.

MASQ-D30: The cut-off score of 19 on the General Distress (GD) dimension, which discriminated the ROM reference group from the ROM patient group, had a sensitivity and specificity of 90%. For the cut-off of 23 on the Anhedonic Depression dimension, the sensitivity and specificity were only 80%. The cut-off score of 18 on the Anxious Arousal dimension, discriminating health from disease, had a sensitivity and specificity of 96%. The AUC values showed that all three scales performed well in discriminating between outpatients and non-patients. The most discriminating subscale was Anxious Arousal (AUC=0.99), followed by General Distress (AUC=0.96) and Anhedonic Depression (AUC=0.88). See Figure 1 for the discriminative power of the General Distress score.

SF-36: The cut-off point of the Mental Health score, which discriminated the ROM reference group from the ROM patient group, was 63, with a sensitivity and specificity of 89%. The AUC values showed that all SF-36 subscales performed well in making a distinction between patients and non-patients. The discriminating performance of Mental Health was excellent (AUC=0.95). The next best discriminating subscales were Social Functioning (AUC=0.92) and Vitality (AUC=0.92). The Bodily Pain and Physical Functioning scales showed the least distinctiveness, but they were still adequate, and are therefore still clinically useful. The discriminative power of General Mental Health is presented in Figure 1.

DAPP-SF: The cut-off point of the Identity Problems score, which discriminated the ROM reference group from the ROM patient group, was 2.08, with a sensitivity and specificity of 83%. The cut-off point of the Oppositionality score was 2.22 with a sensitivity and specificity of 73%. The discriminating performance of the DAPP-SF was moderate. The AUC values showed that 11 subscales performed well in distinguishing between patients and non-patients. The best performing subscale was Identity Problems (AUC=0.90), followed by Affective Lability (AUC=0.90) and Anxiousness (AUC=0.90). Seven subscales showed no clinically useful discriminatory power, with AUC values ranging from 0.53 to 0.60. All scales might perform better in the specific subpopulation of patients with personality disorders. As an example, the distributions of Oppositionality in the ROM reference group and the ROM patient group are presented in Figure 1. (This subscale was selected because it showed substantial interperson variability.)

## Discussion

We reported reference values (95^th^ percentiles) for the generic instruments BSI, MASQ-D30, SF-36 and DAPP-SF in large samples from 'healthy' and ‘psychiatrically ill’ populations. The internal consistency of the total score and subscale scores of the four generic instruments was consistently high. In the two samples, the expected differences in mean scores were confirmed, validating the clinical application of the ROC cut-off values or the 95^th^ percentile scores (or 5^th^ percentile for the SF-36). A clear gender difference in reference values was observed, with women showing higher values than men. It is remarkable that “healthy” men and women differed, and that the gender-specific distributions of the generic scales overlapped but did not coincide. Our data suggested that the degree of overlap between the sexes was not negligible, and that sex-specific reference values would increase the precision of the assessment of the clinical state of psychiatric outpatients. Advancing age was associated with more symptoms of Axis I psychopathology. Consequently, to be regarded as recovered, a young man would need to have lower scores on generic scales than would an older woman.

ROC analyses showed good discriminative power for the BSI, MASQ-D30, and SF-36 but not for the DAPP-SF subscales. The former three instruments address Axis-I psychopathology or distress, whereas the DAPP-SF measures Axis-II personality traits that are rather stable and less affected by psychopathology and treatment. The higher AUC values represent the more state-like than trait-like characteristics of the BSI, MASQ-D30, and SF-36, compared to the DAPP-SF.

The high internal consistency of the BSI, MASQ-D30, SF-36, and DAPP-SF are in accordance with previous studies [11,14,18,19,23,34]. Subscale means for the ROM reference group were somewhat lower than reported in previous studies of general population samples for the BSI [11,19]. In addition, they were slightly higher than in most [15,34-37] but not all [38] SF-36 studies and lower than in a DAPP-SF study [18]. Regarding the ROM patient group, means for the BSI, SF-36, and DAPP-SF approximated previously reported values in most clinical populations [11,15,19,23]. Previously, reference values subcategorized by gender and age have only been reported for the SF-36 [21,22,25]. Given that the assessment results for our ROM instruments generally had skewed distributions with a long tail toward the extreme values (i.e., lower in the case of the SF-36), we preferred percentile scores rather than means and SDs, in contrast to previous studies. For the BSI, ROC cut-off scores approximated cut-off scores with optimal sensitivity, as reported by De Beurs and Zitman (2006). Further, P_95_ reference scores approximated De Beurs and Zitman ‘s cut-off scores with optimal specificity [11].

Reference values derived from the ROM reference and patient groups have different functions. Reference values from the ROM reference and patient groups are important for screening a patient who is considered to have more than mild abnormalities. A precisely defined reference value will allow for the detection of subjects with psychopathology who could benefit from therapy or from referral from primary care to specialized mental health care (and vice versa). For screening purposes, we recommend the use of cut-off scores with a high sensitivity, to be sure that a minimal number of patients with psychopathology get through undetected, although this would result in higher false positives. So, for the purpose of screening, ROC-based cut-offs, 75^th^ percentile scores from the ROM reference group, or 5^th^ percentile scores from the ROM patient group may be appropriate; for the SF-36 this would be represented by the 25^th^ and 95^th^ percentiles, respectively [26]. However, if the consequences of missing the disease are relatively minor, and if the costs of therapy providing for subjects who are wrongfully diagnosed are substantial, a somewhat higher specificity with lower sensitivity may be used [39]. The reference values established in the present study can be used to determine whether a patient’s level of symptoms falls within the normal range of values after treatment (e.g., whether a treated patient is no longer any different from normal controls with respect to the level of depressive symptoms). These reference values are to be used to determine treatment goals.

Normality can be defined statistically or medically. The statistical model is based on the distribution of scores from the general population (including all individuals) and on deviation from the mean. The middle range of scores of the normal distribution is considered as normal (within 2 SD of the mean), and extreme high or low scores are considered deviant. The medical model considers psychopathology and normality (i.e. absence of psychopathology) in absolute terms. It excludes individuals with a disorder from a reference group [40]. In our study we chose the statistical approach and therefore included all non-consulting individuals, both with and without (sub clinical) symptoms. So, there are different viewpoints as to whether the general population should consist of non-treated subjects or whether it should be more restricted (i.e., only including subjects without psychiatric diagnoses). We have chosen for the former definition, because we tested generic instruments which are not confined to a single DSM-IV diagnosis. If we had excluded 122 (9.5%) subjects with a MINI-diagnosis from the main analysis, we think that the reference values would have been too strict. Nevertheless, we have already shown above that the reference values were not affected to any large extent by our inclusive methodology.

The present study has several strengths. The ROM reference group was sufficiently large, clearly defined, and similar to the ROM patient group with respect to age, gender, and level of urbanization. These non-consulting GP patients were highly representative of the general population, given the extremely high GP registration percentage. This was further illustrated by the fact that sufficient psychiatric symptoms were reported by approximately 10% of the population-based reference group to the point of warranting a DSM-IV diagnosis, which is in line with a Dutch (NEMESIS;Bijl et al. 1998) comorbidity study. Stratification of the ROM reference group into more homogeneous gender- and age-subsets resulted in a better differentiation of reference values. Assessment and analytical procedures were standardized and of high quality, similar to the ones used for the ROM patients.

Limitations of our study that should be mentioned include the high non-response (63.2%) in the ROM reference group, which may have resulted in bias due to selection. Some populations (i.e., younger males with full-time employment) may have been underrepresented. We believe that this may have resulted in a slight under-representation of the healthiest subjects, overly conservative estimates of the discriminative power of the instruments, slightly low percentile scores, and slightly high cut-off points for the transition from healthy to psychiatrically ill. At the same time, analyses of data from the ROM reference group without the 10.9% of subjects with a MINI diagnosis did not substantially alter our findings, suggesting that our reference values were fairly robust. As no information was available for non-responders and excluded individuals, they could not be compared with the ROM reference group for demographic variables. Furthermore, ethnic and cultural differences were not considered. Therefore, our reference values for the Dutch general population may not directly apply to other ethnic or cultural groups. Likewise, reference values for children and the elderly remain to be assessed. Another issue concerns the use of the DAPP-SF for the assessment of dysfunctional personality traits. It has been suggested that the limited validity of self-report instruments for assessing personality pathology is particularly relevant in clinical populations [41], especially among depressed [42] and psychotic patients [43]. Finally, it is important to recognize the limitations of population-based reference values. They should not be interpreted too rigidly.

## Conclusion

This large-scale population-based study provides reference values for the BSI, MASQ-D30, SF-36, and DAPP-SF. These reference values are essential for use in clinical psychiatry care. The scales are commonly incorporated in the comprehensive set of generic ROM instruments and they can be administered with every patient with psychiatric disorders for the purpose of routine screening, referral, and treatment. This set of four scales thoroughly covers general psychopathology, mood- and anxiety disorders (which represent 80% of psychiatric disorders), personality disorders, and quality of life. ROM reference values inform therapists and patients on the severity of the complaints at intake, and the waxing and waning of symptoms over the course of treatment. Furthermore, they enable research of the effectiveness of treatments in everyday clinical practice and managers can use them for benchmarking.

## Competing interests

The authors declare that they have no competing interests.

## Authors' contributions

YSvM contributed to the study design and coordination of data collection, undertook the statistical analyses, and drafted the manuscript. IC contributed to the study design, the coordination of data collection, and assisted in editing the manuscript. FZ conceived of the study, contributed to its design, obtained funding, and contributed to interpretation of data analyses and editing of the manuscript. AvH contributed to interpretation of data analyses and editing of the manuscript. MdW played a key role in the recruitment of the reference group. MvN participated in the study design. EG contributed to the conception and design of the study, assisted in the statistical analyses and interpretation, and assisted in the writing of the manuscript. All authors read and approved the final manuscript.

## Pre-publication history

The pre-publication history for this paper can be accessed here:

http://www.biomedcentral.com/1471-244X/12/203/prepub

## Supplementary Material

Additional file 1 **Table S1.** Percentile scores and mean values in the ROM reference (n=1294) and patient (n=5269) groups for the subscales and total score of the **Brief Symptom Inventory (BSI)**. **Table S2.**Percentile scores and mean values in the ROM reference (n=1294) and patient (n=5269) groups for the subscales and total score of the **Mood & Anxiety Symptom Questionnaire-30 (MASQ-D30). Table S3.** Percentile scores and mean values in the ROM reference (n=1294) and patient (n=5269) groups for the subscales and total score of the **Short Form 36 (SF36). Table S4.** Percentile scores and mean values in the ROM reference (n=635) and patient (n=5035) groups for the subscales and total score of the **Dimensional Assessment of Personality Pathology – short form (DAPP-SF).** Click here for file
