# Supplementary material for: Reference values for generic instruments used in routine outcome monitoring: the leiden routine outcome monitoring study
Source: BMC Psychiatry. 2012 Nov 21;12:203. doi: 10.1186/1471-244X-12-203 (PMC3551660; doi:10.1186/1471-244X-12-203)
Supplement: Additional file 1 — Table S1. Percentile scores and mean values in the ROM reference (n=1294) and patient (n=5269) groups for the subscales and total score of the Brief Symptom Inventory (BSI). Table S2.Percentile scores and mean values in the ROM reference (n=1294) and patient (n=5269) groups for the subscales and total score of the Mood & Anxiety Symptom Questionnaire-30 (MASQ-D30). Table S3. Percentile scores and mean values in the ROM reference (n=1294) and patient (n=5269) groups for the subscales and total score of the Short Form 36 (SF36). Table S4. Percentile scores and mean values in the ROM reference (n=635) and patient (n=5035) groups for the subscales and total score of the Dimensional Assessment of Personality Pathology – short form (DAPP-SF). [file 1471-244X-12-203-S1.pdf]

**Supplementary Table 1.**

Percentile scores and mean values in the ROM reference (n=1294) and patient (n=5269) groups for the subscales and total score of the **Brief Symptom Inventory (BSI)**.

| | ROM reference group (n=1294) | | | | | | ROM patient group (n=5269) | | | | | |
|---|---|---|---|---|---|---|---|---|---|---|---|---|
| | $P_5$ | $P_{25}$ | $P_{50}$ (median) | $P_{75}$ | $P_{95}$ | Mean ± SD | $P_5$ | $P_{25}$ | $P_{50}$ (median) | $P_{75}$ | $P_{95}$ | Mean ± SD |
| ***Somatization (SOM)*** | | | | | | | | | | | | |
| All participants | 0.00 | 0.00 | 0.00 | 0.29 | 0.71 | 0.17 ± 0.28 | 0.00 | 0.43 | 0.86 | 1.43 | 2.71 | 1.03 ± 0.83 |
| - Women aged 18-40 yr | 0.00 | 0.00 | 0.14 | 0.29 | 0.86 | 0.20 ± 0.31 | 0.00 | 0.43 | 0.86 | 1.57 | 2.71 | 1.06 ± 0.85 |
| - Women aged 41-65 yr | 0.00 | 0.00 | 0.14 | 0.29 | 0.86 | 0.20 ± 0.29 | 0.14 | 0.43 | 0.86 | 1.57 | 2.71 | 1.09 ± 0.81 |
| - Men aged 18-40 yr | 0.00 | 0.00 | 0.00 | 0.14 | 0.44 | 0.11 ± 0.19 | 0.00 | 0.29 | 0.71 | 1.43 | 2.57 | 0.94 ± 0.79 |
| - Men aged 41-65 yr | 0.00 | 0.00 | 0.00 | 0.14 | 0.57 | 0.11 ± 0.23 | 0.00 | 0.29 | 0.71 | 1.43 | 2.68 | 0.96 ± 0.82 |
| ***Obsessive-Compulsive (O-C)*** | | | | | | | | | | | | |
| All participants | 0.00 | 0.00 | 0.17 | 0.50 | 1.17 | 0.35 ± 0.42 | 0.33 | 1.00 | 1.67 | 2.33 | 3.33 | 1.67 ± 0.95 |
| - Women aged 18-40 yr | 0.00 | 0.00 | 0.33 | 0.50 | 1.17 | 0.38 ± 0.44 | 0.33 | 1.00 | 1.67 | 2.33 | 3.33 | 1.68 ± 0.95 |
| - Women aged 41-65 yr | 0.00 | 0.00 | 0.17 | 0.50 | 1.25 | 0.37 ± 0.45 | 0.27 | 0.83 | 1.67 | 2.33 | 3.17 | 1.64 ± 0.92 |
| - Men aged 18-40 yr | 0.00 | 0.00 | 0.17 | 0.50 | 1.17 | 0.32 ± 0.37 | 0.17 | 0.83 | 1.50 | 2.33 | 3.33 | 1.64 ± 0.95 |
| - Men aged 41-65 yr | 0.00 | 0.00 | 0.17 | 0.50 | 1.00 | 0.29 ± 0.37 | 0.22 | 1.00 | 1.67 | 2.50 | 3.50 | 1.73 ± 0.98 |
| ***Interpersonal Sensitivity (I-S)*** | | | | | | | | | | | | |
| All participants | 0.00 | 0.00 | 0.00 | 0.50 | 1.00 | 0.29 ± 0.42 | 0.00 | 0.75 | 1.50 | 2.25 | 3.50 | 1.56 ± 1.04 |
| - Women aged 18-40 yr | 0.00 | 0.00 | 0.25 | 0.50 | 1.25 | 0.35 ± 0.46 | 0.25 | 0.75 | 1.63 | 2.50 | 3.75 | 1.74 ± 1.09 |
| - Women aged 41-65 yr | 0.00 | 0.00 | 0.25 | 0.50 | 1.25 | 0.31 ± 0.44 | 0.00 | 0.69 | 1.25 | 2.00 | 3.50 | 1.45 ± 1.01 |
| - Men aged 18-40 yr | 0.00 | 0.00 | 0.00 | 0.25 | 1.00 | 0.23 ± 0.34 | 0.00 | 0.75 | 1.25 | 2.00 | 3.25 | 1.48 ± 0.98 |
| - Men aged 41-65 yr | 0.00 | 0.00 | 0.00 | 0.25 | 0.75 | 0.17 ± 0.34 | 0.00 | 0.50 | 1.25 | 2.00 | 3.25 | 1.35 ± 0.97 |
| ***Depression (DEP)*** | | | | | | | | | | | | |
| All participants | 0.00 | 0.00 | 0.00 | 0.33 | 0.83 | 0.20 ± 0.34 | 0.17 | 0.83 | 1.67 | 2.50 | 3.50 | 1.68 ± 1.01 |
| - Women aged 18-40 yr | 0.00 | 0.00 | 0.00 | 0.33 | 0.83 | 0.22 ± 0.34 | 0.17 | 0.83 | 1.67 | 2.50 | 3.50 | 1.71 ± 1.05 |
| - Women aged 41-65 yr | 0.00 | 0.00 | 0.00 | 0.33 | 0.83 | 0.22 ± 0.37 | 0.17 | 0.83 | 1.50 | 2.50 | 3.33 | 1.66 ± 1.01 |
| - Men aged 18-40 yr | 0.00 | 0.00 | 0.00 | 0.17 | 0.68 | 0.17 ± 0.30 | 0.17 | 0.83 | 1.67 | 2.33 | 3.33 | 1.66 ± 0.96 |
| - Men aged 41-65 yr | 0.00 | 0.00 | 0.00 | 0.17 | 0.67 | 0.14 ± 0.31 | 0.17 | 0.83 | 1.50 | 2.42 | 3.50 | 1.68 ± 0.99 |
| ***Anxiety (ANX)*** | | | | | | | | | | | | |
| All participants | 0.00 | 0.00 | 0.17 | 0.33 | 0.83 | 0.22 ± 0.34 | 0.17 | 0.83 | 1.33 | 2.17 | 3.33 | 1.49 ± 0.94 |
| - Women aged 18-40 yr | 0.00 | 0.00 | 0.17 | 0.33 | 1.00 | 0.27 ± 0.39 | 0.17 | 0.83 | 1.33 | 2.17 | 3.33 | 1.53 ± 0.95 |
| - Women aged 41-65 yr | 0.00 | 0.00 | 0.17 | 0.33 | 0.92 | 0.23 ± 0.36 | 0.17 | 0.83 | 1.33 | 2.17 | 3.33 | 1.49 ± 0.94 |
| - Men aged 18-40 yr | 0.00 | 0.00 | 0.17 | 0.33 | 0.67 | 0.19 ± 0.25 | 0.17 | 0.67 | 1.33 | 2.00 | 3.17 | 1.42 ± 0.92 |
| - Men aged 41-65 yr | 0.00 | 0.00 | 0.00 | 0.17 | 0.67 | 0.17 ± 0.29 | 0.17 | 0.67 | 1.33 | 2.00 | 3.33 | 1.49 ± 0.94 |
| ***Hostility (HOS)*** | | | | | | | | | | | | |
| All participants | 0.00 | 0.00 | 0.20 | 0.20 | 0.80 | 0.20 ± 0.29 | 0.00 | 0.20 | 0.80 | 1.40 | 2.80 | 0.94 ± 0.86 |
| - Women aged 18-40 yr | 0.00 | 0.00 | 0.20 | 0.20 | 1.00 | 0.25 ± 0.36 | 0.00 | 0.40 | 0.80 | 1.60 | 3.00 | 1.07 ± 0.90 |
| - Women aged 41-65 yr | 0.00 | 0.00 | 0.20 | 0.20 | 0.60 | 0.18 ± 0.24 | 0.00 | 0.20 | 0.60 | 1.00 | 2.20 | 0.73 ± 0.73 |
| - Men aged 18-40 yr | 0.00 | 0.00 | 0.20 | 0.20 | 0.60 | 0.17 ± 0.24 | 0.00 | 0.40 | 0.80 | 1.40 | 2.80 | 0.97 ± 0.86 |
| - Men aged 41-65 yr | 0.00 | 0.00 | 0.20 | 0.20 | 0.60 | 0.17 ± 0.23 | 0.00 | 0.20 | 0.60 | 1.20 | 2.60 | 0.90 ± 0.84 |
| ***Phobic Anxiety (PHOB)*** | | | | | | | | | | | | |
| All participants | 0.00 | 0.00 | 0.00 | 0.20 | 0.60 | 0.11 ± 0.23 | 0.00 | 0.40 | 1.00 | 1.60 | 3.00 | 1.15 ± 0.93 |
| - Women aged 18-40 yr | 0.00 | 0.00 | 0.00 | 0.20 | 0.60 | 0.11 ± 0.23 | 0.00 | 0.40 | 1.00 | 1.80 | 3.20 | 1.19 ± 0.96 |
| - Women aged 41-65 yr | 0.00 | 0.00 | 0.00 | 0.20 | 0.80 | 0.13 ± 0.26 | 0.00 | 0.40 | 0.80 | 1.60 | 3.00 | 1.12 ± 0.94 |
| - Men aged 18-40 yr | 0.00 | 0.00 | 0.00 | 0.00 | 0.40 | 0.07 ± 0.16 | 0.00 | 0.40 | 1.00 | 1.65 | 3.00 | 1.15 ± 0.91 |
| - Men aged 41-65 yr | 0.00 | 0.00 | 0.00 | 0.00 | 0.60 | 0.10 ± 0.23 | 0.00 | 0.40 | 0.80 | 1.60 | 2.80 | 1.09 ± 0.89 |
| ***Paranoid Ideation (PAR)*** | | | | | | | | | | | | |
| All participants | 0.00 | 0.00 | 0.00 | 0.40 | 0.80 | 0.23 ± 0.35 | 0.00 | 0.40 | 1.00 | 1.80 | 3.00 | 1.15 ± 0.94 |
| - Women aged 18-40 yr | 0.00 | 0.00 | 0.20 | 0.40 | 0.82 | 0.25 ± 0.38 | 0.00 | 0.40 | 1.00 | 1.80 | 3.00 | 1.20 ± 0.96 |
| - Women aged 41-65 yr | 0.00 | 0.00 | 0.20 | 0.40 | 0.80 | 0.23 ± 0.33 | 0.00 | 0.40 | 0.80 | 1.60 | 2.80 | 1.07 ± 0.90 |
| - Men aged 18-40 yr | 0.00 | 0.00 | 0.20 | 0.40 | 0.80 | 0.22 ± 0.31 | 0.00 | 0.40 | 1.00 | 1.80 | 3.00 | 1.14 ± 0.93 |
| - Men aged 41-65 yr | 0.00 | 0.00 | 0.00 | 0.20 | 0.80 | 0.19 ± 0.34 | 0.00 | 0.40 | 1.00 | 1.80 | 3.00 | 1.15 ± 0.94 |
| ***Psychoticism (PSY)*** | | | | | | | | | | | | |
| All participants | 0.00 | 0.00 | 0.00 | 0.20 | 0.80 | 0.14 ± 0.28 | 0.20 | 0.60 | 1.20 | 1.80 | 2.80 | 1.23 ± 0.81 |
| - Women aged 18-40 yr | 0.00 | 0.00 | 0.00 | 0.20 | 0.80 | 0.16 ± 0.29 | 0.20 | 0.60 | 1.20 | 1.80 | 2.80 | 1.29 ± 0.85 |
| - Women aged 41-65 yr | 0.00 | 0.00 | 0.00 | 0.20 | 0.80 | 0.15 ± 0.28 | 0.00 | 0.60 | 1.00 | 1.60 | 2.60 | 1.13 ± 0.79 |
| - Men aged 18-40 yr | 0.00 | 0.00 | 0.00 | 0.20 | 0.80 | 0.14 ± 0.28 | 0.20 | 0.60 | 1.20 | 1.80 | 2.80 | 1.26 ± 0.79 |
| - Men aged 41-65 yr | 0.00 | 0.00 | 0.00 | 0.20 | 0.60 | 0.11 ± 0.25 | 0.20 | 0.60 | 1.00 | 1.60 | 2.60 | 1.19 ± 0.78 |
| ***BSI total score*** | | | | | | | | | | | | |
| All participants | 0.00 | 0.06 | 0.13 | 0.28 | 0.68 | 0.21 ± 0.25 | 0.34 | 0.79 | 1.23 | 1.75 | 2.66 | 1.33 ± 0.71 |
| - Women aged 18-40 yr | 0.00 | 0.06 | 0.17 | 0.34 | 0.72 | 0.25 ± 0.27 | 0.34 | 0.83 | 1.30 | 1.85 | 2.74 | 1.38 ± 0.73 |
| - Women aged 41-65 yr | 0.00 | 0.06 | 0.15 | 0.30 | 0.78 | 0.23 ± 0.25 | 0.30 | 0.75 | 1.19 | 1.72 | 2.55 | 1.28 ± 0.69 |
| - Men aged 18-40 yr | 0.00 | 0.06 | 0.13 | 0.21 | 0.55 | 0.18 ± 0.20 | 0.34 | 0.79 | 1.21 | 1.72 | 2.58 | 1.29 ± 0.68 |
| - Men aged 41-65 yr | 0.00 | 0.02 | 0.09 | 0.23 | 0.51 | 0.16 ± 0.22 | 0.31 | 0.75 | 1.21 | 1.68 | 2.70 | 1.29 ± 0.72 |

ROM: Routine outcome monitoring.

**Supplementary Table 2.**
Percentile scores and mean values in the ROM reference (n=1294) and patient (n=5269) groups for the subscales and total score of the **Mood & Anxiety Symptom Questionnaire-30 (MASQ-D30).**

| | ROM reference group (n=1294) | | | | | | ROM patient group (n=5269) | | | | | |
|---|---|---|---|---|---|---|---|---|---|---|---|---|
| | **P5** | **P25** | **P50 (median)** | **P75** | **P95** | **Mean ± SD** | **P5** | **P25** | **P50 (median)** | **P75** | **P95** | **Mean ± SD** |
| ***General distress (GD)*** | | | | | | | | | | | | |
| All participants | 10 | 11 | 12 | 15 | 23 | 13.8 ± 4.4 | 17 | 23 | 28 | 33 | 40 | 28.1 ± 6.9 |
| - Women aged 18-40 yr | 10 | 11 | 13 | 17 | 25 | 14.7 ± 5.0 | 17 | 24 | 29 | 34 | 40 | 28.6 ± 7.0 |
| - Women aged 41-65 yr | 10 | 11 | 12 | 15 | 23 | 13.7 ± 4.4 | 17 | 23 | 28 | 33 | 40 | 28.1 ± 6.9 |
| - Men aged 18-40 yr | 10 | 11 | 12 | 15 | 21 | 13.2 ± 3.7 | 16 | 23 | 27 | 32 | 39 | 27.3 ± 6.6 |
| - Men aged 41-65 yr | 10 | 10 | 12 | 14 | 20 | 12.8 ± 3.8 | 17 | 23 | 27 | 32 | 40 | 27.7 ± 6.8 |
| ***Anhedonic depression (AD)*** | | | | | | | | | | | | |
| All participants | 10 | 14 | 17 | 22 | 29 | 18.4 ± 5.8 | 17 | 24 | 31 | 37 | 44 | 30.7 ± 8.3 |
| - Women aged 18-40 yr | 11 | 14 | 17 | 22 | 28 | 18.3 ± 5.5 | 17 | 24 | 30 | 37 | 44 | 30.4 ± 8.5 |
| - Women aged 41-65 yr | 11 | 15 | 18 | 23 | 32 | 19.4 ± 6.2 | 18 | 24 | 31 | 37 | 44 | 30.9 ± 8.4 |
| - Men aged 18-40 yr | 9 | 13 | 16 | 20 | 28 | 17.0 ± 5.4 | 18 | 25 | 31 | 37 | 44 | 30.9 ± 8.0 |
| - Men aged 41-65 yr | 11 | 14 | 17 | 22 | 29 | 18.6 ± 5.6 | 17 | 25 | 31 | 37 | 44 | 31.1 ± 8.2 |
| ***Anxious arousal (AA)*** | | | | | | | | | | | | |
| All participants | 10 | 10 | 11 | 13 | 17 | 11.9 ± 3.0 | 18 | 26 | 31 | 37 | 43 | 31.3 ± 7.5 |
| - Women aged 18-40 yr | 10 | 10 | 11 | 13 | 18 | 12.3 ± 3.5 | 18 | 26 | 31 | 37 | 43 | 31.2 ± 7.6 |
| - Women aged 41-65 yr | 10 | 10 | 11 | 13 | 19 | 12.2 ± 3.1 | 18 | 26 | 31 | 37 | 44 | 31.1 ± 7.5 |
| - Men aged 18-40 yr | 10 | 10 | 10 | 12 | 15 | 11.3 ± 2.0 | 19 | 26 | 32 | 36 | 43 | 31.3 ± 7.3 |
| - Men aged 41-65 yr | 10 | 10 | 10 | 12 | 16 | 11.3 ± 2.2 | 18 | 27 | 32 | 37 | 44 | 31.6 ± 7.5 |

ROM: Routine outcome monitoring.

**Supplementary Table 3.**
Percentile scores and mean values in the ROM reference (n=1294) and patient (n=5269) groups for the subscales and total score of the **Short Form 36 (SF36).**

| | ROM reference group (n=1294) | | | | | | ROM patient group (n=5269) | | | | | |
|---|---|---|---|---|---|---|---|---|---|---|---|---|
| | $P_5$ | $P_{25}$ | $P_{50}$ (median) | $P_{75}$ | $P_{95}$ | Mean ± SD | $P_5$ | $P_{25}$ | $P_{50}$ (median) | $P_{75}$ | $P_{95}$ | Mean ± SD |
| ***Physical Functioning*** | | | | | | | | | | | | |
| All participants | 65 | 90 | 100 | 100 | 100 | 92.6 ± 14.2 | 25 | 60 | 80 | 95 | 100 | 74.8 ± 23.7 |
| - Women aged 18-40 yr | 70 | 95 | 100 | 100 | 100 | 93.8 ± 12.4 | 30 | 60 | 85 | 95 | 100 | 76.3 ± 23.1 |
| - Women aged 41-65 yr | 53 | 85 | 95 | 100 | 100 | 89.4 ± 17.0 | 18 | 50 | 73 | 90 | 100 | 67.8 ± 25.2 |
| - Men aged 18-40 yr | 80 | 95 | 100 | 100 | 100 | 96.9 ± 7.8 | 40 | 70 | 90 | 100 | 100 | 81.3 ± 20.5 |
| - Men aged 41-65 yr | 55 | 90 | 95 | 100 | 100 | 91.1 ± 15.9 | 25 | 55 | 80 | 90 | 100 | 73.1 ± 24.0 |
| ***Role-physical*** | | | | | | | | | | | | |
| All participants | 5 | 100 | 100 | 100 | 100 | 87.0 ± 27.2 | 0 | 0 | 25 | 75 | 100 | 37.2 ± 39.7 |
| - Women aged 18-40 yr | 25 | 75 | 100 | 100 | 100 | 87.0 ± 26.4 | 0 | 0 | 25 | 75 | 100 | 38.8 ± 40.1 |
| - Women aged 41-65 yr | 0 | 75 | 100 | 100 | 100 | 84.4 ± 31.0 | 0 | 0 | 0 | 50 | 100 | 30.9 ± 38.7 |
| - Men aged 18-40 yr | 25 | 100 | 100 | 100 | 100 | 90.9 ± 22.6 | 0 | 0 | 25 | 75 | 100 | 44.2 ± 40.2 |
| - Men aged 41-65 yr | 25 | 70 | 100 | 100 | 100 | 87.0 ± 26.3 | 0 | 0 | 25 | 75 | 100 | 33.8 ± 38.0 |
| ***Bodily Pain*** | | | | | | | | | | | | |
| All participants | 54 | 78 | 90 | 100 | 100 | 86.4 ± 17.6 | 20 | 45 | 67 | 90 | 100 | 65.9 ± 27.5 |
| - Women aged 18-40 yr | 54 | 78 | 90 | 100 | 100 | 84.2 ± 18.2 | 20 | 45 | 67 | 90 | 100 | 65.7 ± 26.9 |
| - Women aged 41-65 yr | 45 | 78 | 90 | 100 | 100 | 84.8 ± 19.2 | 10 | 45 | 57 | 90 | 100 | 61.3 ± 28.4 |
| - Men aged 18-40 yr | 67 | 90 | 100 | 100 | 100 | 91.4 ± 13.6 | 22 | 57 | 78 | 100 | 100 | 72.0 ± 26.0 |
| - Men aged 41-65 yr | 57 | 80 | 90 | 100 | 100 | 87.9 ± 16.2 | 20 | 45 | 67 | 90 | 100 | 65.7 ± 28.2 |
| ***Social Functioning*** | | | | | | | | | | | | |
| All participants | 63 | 88 | 100 | 100 | 100 | 89.9 ± 15.6 | 0 | 25 | 50 | 63 | 88 | 44.8 ± 26.1 |
| - Women aged 18-40 yr | 50 | 75 | 100 | 100 | 100 | 88.2 ± 16.8 | 0 | 25 | 50 | 63 | 88 | 44.5 ± 26.2 |
| - Women aged 41-65 yr | 56 | 88 | 100 | 100 | 100 | 88.7 ± 16.1 | 0 | 25 | 38 | 63 | 88 | 42.4 ± 25.9 |
| - Men aged 18-40 yr | 63 | 88 | 100 | 100 | 100 | 93.8 ± 12.3 | 0 | 25 | 50 | 63 | 88 | 48.3 ± 26.1 |
| - Men aged 41-65 yr | 63 | 88 | 100 | 100 | 100 | 90.9 ± 14.9 | 0 | 25 | 50 | 63 | 88 | 44.9 ± 25.6 |
| ***Mental health*** | | | | | | | | | | | | |
| All participants | 56 | 72 | 80 | 88 | 96 | 79.7 ± 12.3 | 12 | 28 | 40 | 52 | 76 | 41.5 ± 18.2 |
| - Women aged 18-40 yr | 56 | 72 | 80 | 88 | 96 | 78.9 ± 12.2 | 12 | 28 | 40 | 52 | 76 | 42.0 ± 18.2 |
| - Women aged 41-65 yr | 52 | 72 | 80 | 88 | 94 | 78.2 ± 13.3 | 12 | 28 | 40 | 52 | 76 | 41.1 ± 18.8 |
| - Men aged 18-40 yr | 64 | 76 | 84 | 89 | 96 | 81.8 ± 10.8 | 16 | 32 | 40 | 52 | 72 | 41.9 ± 17.2 |
| - Men aged 41-65 yr | 60 | 76 | 84 | 88 | 96 | 81.5 ± 11.6 | 12 | 28 | 40 | 52 | 76 | 40.5 ± 18.5 |
| ***Role-emotional*** | | | | | | | | | | | | |
| All participants | 33 | 100 | 100 | 100 | 100 | 90.4 ± 24.8 | 0 | 0 | 0 | 33 | 100 | 28.2 ± 36.2 |
| - Women aged 18-40 yr | 0 | 100 | 100 | 100 | 100 | 88.5 ± 26.9 | 0 | 0 | 0 | 50 | 100 | 28.4 ± 36.4 |
| - Women aged 41-65 yr | 0 | 100 | 100 | 100 | 100 | 88.4 ± 28.5 | 0 | 0 | 0 | 33 | 100 | 26.0 ± 36.3 |
| - Men aged 18-40 yr | 67 | 100 | 100 | 100 | 100 | 94.3 ± 16.5 | 0 | 0 | 33 | 67 | 100 | 30.3 ± 35.8 |
| - Men aged 41-65 yr | 33 | 100 | 100 | 100 | 100 | 92.9 ± 20.7 | 0 | 0 | 0 | 33 | 100 | 28.7 ± 36.1 |
| ***Vitality*** | | | | | | | | | | | | |
| All participants | 40 | 60 | 70 | 80 | 90 | 68.6 ± 15.3 | 5 | 20 | 35 | 45 | 65 | 34.3 ± 17.8 |
| - Women aged 18-40 yr | 40 | 55 | 70 | 75 | 90 | 66.2 ± 15.0 | 5 | 20 | 35 | 45 | 65 | 34.1 ± 17.6 |
| - Women aged 41-65 yr | 35 | 60 | 70 | 80 | 90 | 68.7 ± 16.3 | 5 | 20 | 30 | 45 | 65 | 32.6 ± 18.1 |
| - Men aged 18-40 yr | 45 | 60 | 70 | 80 | 90 | 70.1 ± 14.0 | 10 | 25 | 35 | 50 | 70 | 36.9 ± 17.3 |
| - Men aged 41-65 yr | 50 | 60 | 70 | 85 | 95 | 71.3 ± 15.1 | 0 | 20 | 35 | 45 | 65 | 33.9 ± 17.8 |
| ***General Health*** | | | | | | | | | | | | |
| All participants | 45 | 65 | 80 | 90 | 100 | 76.2 ± 16.3 | 20 | 35 | 50 | 65 | 90 | 51.6 ± 21.0 |
| - Women aged 18-40 yr | 45 | 68 | 80 | 90 | 100 | 77.0 ± 16.2 | 18 | 35 | 50 | 69 | 90 | 51.6 ± 21.4 |
| - Women aged 41-65 yr | 37 | 65 | 80 | 90 | 100 | 74.9 ± 17.9 | 20 | 35 | 50 | 65 | 85 | 51.2 ± 20.4 |
| - Men aged 18-40 yr | 55 | 70 | 80 | 90 | 100 | 78.8 ± 13.4 | 20 | 40 | 50 | 70 | 90 | 53.3 ± 20.8 |
| - Men aged 41-65 yr | 40 | 65 | 75 | 85 | 100 | 74.1 ± 16.0 | 15 | 35 | 50 | 65 | 85 | 49.9 ± 20.9 |

ROM: Routine outcome monitoring.
Note: in the SF-36  a higher score means better functioning.

**Table 4 suppl.**

Percentile scores and mean values in the ROM reference (n=635) and patient (n=5035) groups for the subscales and total score of the **Dimensional Assessment of Personality Pathology – short form (DAPP-SF).**

| | ROM reference group (n=635) | | | | | | ROM patient group (n=5035) | | | | | |
|---|---|---|---|---|---|---|---|---|---|---|---|---|
| | $P_5$ | $P_{25}$ | $P_{50}$ (median) | $P_{75}$ | $P_{95}$ | Mean ± SD | $P_5$ | $P_{25}$ | $P_{50}$ (median) | $P_{75}$ | $P_{95}$ | Mean ± SD |
| ***Submissiveness*** | | | | | | | | | | | | |
| All participants | 1.13 | 1.50 | 2.00 | 2.50 | 3.50 | 2.10 ± 0.75 | 1.25 | 2.25 | 3.00 | 3.63 | 4.38 | 2.94 ± 0.94 |
| - Women aged 18-40 yr | 1.13 | 1.50 | 2.13 | 2.75 | 3.75 | 2.20 ± 0.82 | 1.38 | 2.50 | 3.13 | 3.75 | 4.50 | 3.09 ± 0.92 |
| - Women aged 41-65 yr | 1.00 | 1.63 | 2.00 | 2.63 | 3.38 | 2.10 ± 0.71 | 1.25 | 2.25 | 2.88 | 3.63 | 4.38 | 2.91 ± 0.96 |
| - Men aged 18-40 yr | 1.13 | 1.63 | 2.00 | 2.44 | 3.26 | 2.10 ± 0.69 | 1.38 | 2.25 | 2.88 | 3.50 | 4.38 | 2.84 ± 0.90 |
| - Men aged 41-65 yr | 1.00 | 1.25 | 1.75 | 2.25 | 3.38 | 1.89 ± 0.71 | 1.25 | 2.00 | 2.75 | 3.38 | 4.25 | 2.71 ± 0.93 |
| ***Cognitive Distortion*** | | | | | | | | | | | | |
| All participants | 1.00 | 1.00 | 1.17 | 1.50 | 2.33 | 1.36 ± 0.51 | 1.00 | 1.50 | 2.33 | 3.00 | 4.17 | 2.36 ± 0.96 |
| - Women aged 18-40 yr | 1.00 | 1.00 | 1.17 | 1.50 | 2.33 | 1.38 ± 0.56 | 1.00 | 1.67 | 2.33 | 3.17 | 4.17 | 2.40 ± 0.97 |
| - Women aged 41-65 yr | 1.00 | 1.00 | 1.17 | 1.50 | 2.33 | 1.35 ± 0.45 | 1.00 | 1.50 | 2.17 | 2.83 | 4.00 | 2.23 ± 0.93 |
| - Men aged 18-40 yr | 1.00 | 1.00 | 1.17 | 1.50 | 2.68 | 1.36 ± 0.57 | 1.00 | 1.67 | 2.33 | 3.17 | 4.17 | 2.44 ± 0.96 |
| - Men aged 41-65 yr | 1.00 | 1.00 | 1.17 | 1.50 | 2.17 | 1.31 ± 0.41 | 1.00 | 1.50 | 2.33 | 3.00 | 4.00 | 2.34 ± 0.95 |
| ***Identity Problems*** | | | | | | | | | | | | |
| All participants | 1.00 | 1.00 | 1.33 | 1.83 | 2.70 | 1.54 ± 0.59 | 1.33 | 2.33 | 3.17 | 3.83 | 4.67 | 3.12 ± 1.02 |
| - Women aged 18-40 yr | 1.00 | 1.17 | 1.50 | 1.83 | 2.68 | 1.61 ± 0.58 | 1.33 | 2.50 | 3.33 | 4.00 | 4.67 | 3.20 ± 1.00 |
| - Women aged 41-65 yr | 1.00 | 1.00 | 1.33 | 1.83 | 2.83 | 1.54 ± 0.61 | 1.17 | 2.17 | 3.17 | 3.83 | 4.67 | 3.00 ± 1.04 |
| - Men aged 18-40 yr | 1.00 | 1.00 | 1.33 | 1.83 | 3.02 | 1.55 ± 0.61 | 1.33 | 2.50 | 3.33 | 4.00 | 4.67 | 3.18 ± 1.00 |
| - Men aged 41-65 yr | 1.00 | 1.00 | 1.17 | 1.67 | 2.50 | 1.40 ± 0.52 | 1.17 | 2.33 | 3.17 | 3.83 | 4.50 | 3.02 ± 1.02 |
| ***Affective Lability*** | | | | | | | | | | | | |
| All participants | 1.00 | 1.38 | 1.88 | 2.50 | 3.50 | 2.01 ± 0.76 | 1.63 | 2.63 | 3.38 | 3.88 | 4.63 | 3.24 ± 0.88 |
| - Women aged 18-40 yr | 1.00 | 1.56 | 2.00 | 2.75 | 3.64 | 2.16 ± 0.79 | 1.88 | 2.88 | 3.50 | 4.00 | 4.63 | 3.42 ± 0.85 |
| - Women aged 41-65 yr | 1.00 | 1.50 | 2.00 | 2.63 | 3.63 | 2.11 ± 0.79 | 1.50 | 2.50 | 3.25 | 3.88 | 4.50 | 3.16 ± 0.90 |
| - Men aged 18-40 yr | 1.00 | 1.25 | 1.63 | 2.13 | 3.25 | 1.78 ± 0.64 | 1.63 | 2.50 | 3.25 | 3.75 | 4.50 | 3.14 ± 0.87 |
| - Men aged 41-65 yr | 1.00 | 1.25 | 1.63 | 2.00 | 3.38 | 1.78 ± 0.69 | 1.57 | 2.47 | 3.13 | 3.63 | 4.38 | 3.06 ± 0.85 |
| ***Stimulus Seeking*** | | | | | | | | | | | | |
| All participants | 1.10 | 1.38 | 1.88 | 2.38 | 3.38 | 1.99 ± 0.72 | 1.00 | 1.50 | 2.00 | 2.63 | 3.75 | 2.13 ± 0.81 |
| - Women aged 18-40 yr | 1.00 | 1.50 | 1.88 | 2.25 | 3.64 | 2.00 ± 0.75 | 1.00 | 1.50 | 2.00 | 2.50 | 3.63 | 2.10 ± 0.80 |
| - Women aged 41-65 yr | 1.00 | 1.25 | 1.63 | 2.13 | 2.75 | 1.72 ± 0.56 | 1.00 | 1.38 | 1.75 | 2.25 | 3.13 | 1.85 ± 0.65 |
| - Men aged 18-40 yr | 1.11 | 1.88 | 2.38 | 2.88 | 3.50 | 2.37 ± 0.71 | 1.13 | 1.88 | 2.50 | 3.13 | 4.04 | 2.51 ± 0.90 |
| - Men aged 41-65 yr | 1.13 | 1.38 | 2.00 | 2.50 | 3.38 | 2.02 ± 0.70 | 1.00 | 1.63 | 2.06 | 2.63 | 3.63 | 2.15 ± 0.74 |
| ***Compulsivity*** | | | | | | | | | | | | |
| All participants | 1.38 | 2.00 | 2.50 | 3.13 | 4.00 | 2.58 ± 0.77 | 1.38 | 2.13 | 2.88 | 3.63 | 4.50 | 2.89 ± 0.94 |
| - Women aged 18-40 yr | 1.38 | 2.00 | 2.63 | 3.25 | 4.01 | 2.63 ± 0.83 | 1.43 | 2.25 | 2.88 | 3.63 | 4.63 | 2.94 ± 0.95 |
| - Women aged 41-65 yr | 1.25 | 2.00 | 2.56 | 3.16 | 4.00 | 2.59 ± 0.81 | 1.38 | 2.13 | 2.88 | 3.63 | 4.63 | 2.87 ± 0.98 |
| - Men aged 18-40 yr | 1.49 | 2.00 | 2.38 | 2.88 | 3.38 | 2.37 ± 0.59 | 1.25 | 2.13 | 2.75 | 3.38 | 4.29 | 2.75 ± 0.90 |
| - Men aged 41-65 yr | 1.50 | 2.13 | 2.63 | 3.13 | 4.00 | 2.66 ± 0.72 | 1.50 | 2.25 | 3.00 | 3.63 | 4.50 | 2.95 ± 0.91 |
| ***Restricted Expression*** | | | | | | | | | | | | |
| All participants | 1.25 | 1.75 | 2.25 | 2.88 | 3.63 | 2.33 ± 0.75 | 1.75 | 2.63 | 3.25 | 3.88 | 4.63 | 3.23 ± 0.86 |
| - Women aged 18-40 yr | 1.13 | 1.63 | 2.13 | 2.88 | 3.64 | 2.26 ± 0.76 | 1.75 | 2.63 | 3.25 | 3.75 | 4.63 | 3.19 ± 0.85 |
| - Women aged 41-65 yr | 1.25 | 1.75 | 2.25 | 2.88 | 3.63 | 2.33 ± 0.75 | 1.63 | 2.50 | 3.25 | 3.75 | 4.50 | 3.14 ± 0.90 |
| - Men aged 18-40 yr | 1.24 | 1.75 | 2.25 | 2.88 | 3.64 | 2.34 ± 0.74 | 1.88 | 2.75 | 3.38 | 4.00 | 4.75 | 3.37 ± 0.85 |
| - Men aged 41-65 yr | 1.25 | 1.88 | 2.50 | 3.00 | 3.88 | 2.46 ± 0.74 | 1.88 | 2.75 | 3.38 | 3.88 | 4.50 | 3.30 ± 0.82 |
| ***Callousness*** | | | | | | | | | | | | |
| All participants | 1.00 | 1.30 | 1.60 | 2.00 | 2.60 | 1.69 ± 0.50 | 1.00 | 1.30 | 1.70 | 2.10 | 2.90 | 1.77 ± 0.60 |
| - Women aged 18-40 yr | 1.00 | 1.30 | 1.60 | 2.00 | 2.70 | 1.67 ± 0.49 | 1.00 | 1.30 | 1.60 | 2.10 | 2.77 | 1.74 ± 0.56 |
| - Women aged 41-65 yr | 1.00 | 1.10 | 1.40 | 1.80 | 2.30 | 1.50 ± 0.43 | 1.00 | 1.10 | 1.40 | 1.80 | 2.50 | 1.51 ± 0.48 |
| - Men aged 18-40 yr | 1.19 | 1.70 | 2.00 | 2.20 | 2.71 | 1.97 ± 0.49 | 1.10 | 1.60 | 2.00 | 2.50 | 3.30 | 2.10 ± 0.66 |
| - Men aged 41-65 yr | 1.00 | 1.40 | 1.70 | 2.10 | 2.80 | 1.76 ± 0.51 | 1.00 | 1.40 | 1.80 | 2.20 | 2.90 | 1.86 ± 0.58 |
| ***Oppositionality*** | | | | | | | | | | | | |
| All participants | 1.00 | 1.40 | 1.80 | 2.30 | 3.20 | 1.91 ± 0.65 | 1.40 | 2.20 | 2.80 | 3.50 | 4.30 | 2.83 ± 0.89 |
| - Women aged 18-40 yr | 1.10 | 1.40 | 1.80 | 2.30 | 3.20 | 1.96 ± 0.68 | 1.40 | 2.20 | 2.90 | 3.50 | 4.30 | 2.86 ± 0.87 |
| - Women aged 41-65 yr | 1.00 | 1.40 | 1.70 | 2.20 | 2.99 | 1.82 ± 0.59 | 1.20 | 1.90 | 2.60 | 3.30 | 4.20 | 2.62 ± 0.90 |
| - Men aged 18-40 yr | 1.09 | 1.50 | 1.90 | 2.55 | 3.31 | 2.05 ± 0.67 | 1.50 | 2.30 | 3.00 | 3.60 | 4.50 | 3.01 ± 0.89 |
| - Men aged 41-65 yr | 1.00 | 1.40 | 1.70 | 2.20 | 3.10 | 1.85 ± 0.62 | 1.40 | 2.20 | 2.80 | 3.50 | 4.30 | 2.82 ± 0.87 |
| ***Intimacy Problems*** | | | | | | | | | | | | |
| All participants | 1.13 | 1.63 | 2.13 | 2.50 | 3.38 | 2.14 ± 0.67 | 1.13 | 1.75 | 2.38 | 2.88 | 4.00 | 2.42 ± 0.85 |
| - Women aged 18-40 yr | 1.24 | 1.63 | 2.00 | 2.50 | 3.26 | 2.10 ± 0.66 | 1.25 | 1.88 | 2.38 | 2.88 | 4.00 | 2.44 ± 0.83 |
| - Women aged 41-65 yr | 1.25 | 1.88 | 2.25 | 2.66 | 3.75 | 2.35 ± 0.71 | 1.25 | 2.00 | 2.50 | 3.25 | 4.25 | 2.60 ± 0.89 |
| - Men aged 18-40 yr | 1.13 | 1.63 | 2.00 | 2.38 | 3.25 | 2.03 ± 0.61 | 1.13 | 1.63 | 2.13 | 2.75 | 3.68 | 2.25 ± 0.78 |
| - Men aged 41-65 yr | 1.13 | 1.50 | 2.00 | 2.38 | 3.25 | 1.99 ± 0.61 | 1.13 | 1.69 | 2.13 | 2.75 | 3.88 | 2.29 ± 0.82 |
| ***Rejection*** | | | | | | | | | | | | |
| All participants | 1.38 | 1.88 | 2.50 | 3.00 | 3.75 | 2.47 ± 0.76 | 1.13 | 1.63 | 2.25 | 2.88 | 3.75 | 2.31 ± 0.82 |
| - Women aged 18-40 yr | 1.13 | 1.75 | 2.38 | 3.06 | 3.88 | 2.46 ± 0.81 | 1.13 | 1.63 | 2.13 | 2.75 | 3.70 | 2.25 ± 0.79 |
| - Women aged 41-65 yr | 1.13 | 1.63 | 2.25 | 2.88 | 3.48 | 2.28 ± 0.73 | 1.00 | 1.50 | 2.00 | 2.50 | 3.38 | 2.04 ± 0.74 |
| - Men aged 18-40 yr | 1.61 | 2.25 | 2.75 | 3.13 | 3.89 | 2.71 ± 0.67 | 1.25 | 2.00 | 2.63 | 3.13 | 4.00 | 2.58 ± 0.83 |
| - Men aged 41-65 yr | 1.50 | 2.13 | 2.50 | 3.00 | 3.75 | 2.56 ± 0.71 | 1.25 | 1.88 | 2.50 | 3.13 | 3.88 | 2.53 ± 0.82 |

| | | | | | | | | | | | | |
|---|---|---|---|---|---|---|---|---|---|---|---|---|
| ***Anxiousness*** | | | | | | | | | | | | |
| All participants | 1.00 | 1.33 | 1.83 | 2.50 | 3.50 | 2.03 ± 0.81 | 1.67 | 2.67 | 3.50 | 4.00 | 4.83 | 3.37 ± 0.94 |
| - Women aged 18-40 yr | 1.00 | 1.50 | 2.17 | 2.83 | 3.83 | 2.22 ± 0.86 | 1.83 | 3.00 | 3.67 | 4.17 | 4.83 | 3.52 ± 0.90 |
| - Women aged 41-65 yr | 1.00 | 1.33 | 2.00 | 2.67 | 3.33 | 2.06 ± 0.78 | 1.50 | 2.50 | 3.33 | 4.00 | 4.67 | 3.26 ± 0.98 |
| - Men aged 18-40 yr | 1.00 | 1.33 | 1.67 | 2.25 | 3.50 | 1.89 ± 0.75 | 1.67 | 2.75 | 3.50 | 4.00 | 4.67 | 3.35 ± 0.91 |
| - Men aged 41-65 yr | 1.00 | 1.17 | 1.50 | 2.33 | 3.17 | 1.76 ± 0.75 | 1.50 | 2.50 | 3.33 | 3.83 | 4.67 | 3.17 ± 0.95 |
| ***Conduct Problems*** | | | | | | | | | | | | |
| All participants | 1.00 | 1.00 | 1.13 | 1.38 | 2.13 | 1.26 ± 0.37 | 1.00 | 1.00 | 1.25 | 1.63 | 2.63 | 1.43 ± 0.57 |
| - Women aged 18-40 yr | 1.00 | 1.00 | 1.00 | 1.25 | 1.88 | 1.18 ± 0.27 | 1.00 | 1.00 | 1.13 | 1.50 | 2.38 | 1.33 ± 0.48 |
| - Women aged 41-65 yr | 1.00 | 1.00 | 1.00 | 1.13 | 1.63 | 1.13 ± 0.23 | 1.00 | 1.00 | 1.00 | 1.25 | 1.88 | 1.20 ± 0.33 |
| - Men aged 18-40 yr | 1.00 | 1.13 | 1.38 | 1.75 | 2.50 | 1.53 ± 0.49 | 1.00 | 1.25 | 1.63 | 2.25 | 3.25 | 1.80 ± 0.73 |
| - Men aged 41-65 yr | 1.00 | 1.00 | 1.13 | 1.50 | 2.38 | 1.33 ± 0.42 | 1.00 | 1.13 | 1.38 | 1.88 | 2.75 | 1.57 ± 0.60 |
| ***Suspiciousness*** | | | | | | | | | | | | |
| All participants | 1.00 | 1.00 | 1.13 | 1.50 | 2.15 | 1.32 ± 0.46 | 1.00 | 1.38 | 2.00 | 2.88 | 4.00 | 2.18 ± 0.99 |
| - Women aged 18-40 yr | 1.00 | 1.00 | 1.13 | 1.50 | 2.14 | 1.31 ± 0.45 | 1.00 | 1.38 | 2.13 | 3.00 | 4.13 | 2.27 ± 1.02 |
| - Women aged 41-65 yr | 1.00 | 1.00 | 1.13 | 1.25 | 2.11 | 1.25 ± 0.45 | 1.00 | 1.13 | 1.63 | 2.50 | 3.75 | 1.92 ± 0.91 |
| - Men aged 18-40 yr | 1.00 | 1.00 | 1.25 | 1.63 | 2.40 | 1.43 ± 0.50 | 1.00 | 1.50 | 2.25 | 3.00 | 4.13 | 2.33 ± 0.97 |
| - Men aged 41-65 yr | 1.00 | 1.00 | 1.13 | 1.50 | 2.13 | 1.32 ± 0.44 | 1.00 | 1.25 | 2.00 | 2.88 | 4.00 | 2.18 ± 0.99 |
| ***Social Avoidance*** | | | | | | | | | | | | |
| All participants | 1.00 | 1.17 | 1.67 | 2.17 | 3.33 | 1.82 ± 0.73 | 1.17 | 2.17 | 3.00 | 3.83 | 4.67 | 2.98 ± 1.07 |
| - Women aged 18-40 yr | 1.00 | 1.33 | 1.83 | 2.33 | 3.50 | 1.88 ± 0.73 | 1.17 | 2.33 | 3.17 | 4.00 | 4.67 | 3.11 ± 1.06 |
| - Women aged 41-65 yr | 1.00 | 1.17 | 1.67 | 2.17 | 3.33 | 1.80 ± 0.73 | 1.00 | 1.83 | 2.83 | 3.67 | 4.50 | 2.78 ± 1.07 |
| - Men aged 18-40 yr | 1.00 | 1.17 | 1.67 | 2.17 | 3.50 | 1.84 ± 0.77 | 1.17 | 2.33 | 3.17 | 3.83 | 4.67 | 3.05 ± 1.06 |
| - Men aged 41-65 yr | 1.00 | 1.17 | 1.67 | 2.00 | 3.17 | 1.71 ± 0.70 | 1.17 | 2.00 | 2.83 | 3.67 | 4.50 | 2.84 ± 1.04 |
| ***Narcissism*** | | | | | | | | | | | | |
| All participants | 1.00 | 1.63 | 2.13 | 2.63 | 3.50 | 2.18 ± 0.76 | 1.10 | 1.75 | 2.25 | 2.88 | 3.88 | 2.36 ± 0.83 |
| - Women aged 18-40 yr | 1.00 | 1.75 | 2.25 | 3.00 | 3.63 | 2.33 ± 0.79 | 1.25 | 1.88 | 2.50 | 3.00 | 3.88 | 2.47 ± 0.80 |
| - Women aged 41-65 yr | 1.00 | 1.38 | 1.88 | 2.38 | 3.13 | 1.92 ± 0.66 | 1.00 | 1.38 | 1.88 | 2.50 | 3.38 | 1.98 ± 0.73 |
| - Men aged 18-40 yr | 1.25 | 1.88 | 2.50 | 2.88 | 3.89 | 2.43 ± 0.76 | 1.25 | 2.00 | 2.63 | 3.25 | 4.13 | 2.63 ± 0.86 |
| - Men aged 41-65 yr | 1.13 | 1.50 | 2.00 | 2.50 | 3.50 | 2.07 ± 0.73 | 1.00 | 1.63 | 2.25 | 2.88 | 3.88 | 2.31 ± 0.82 |
| ***Insecure Attachment*** | | | | | | | | | | | | |
| All participants | 1.00 | 1.17 | 1.50 | 2.17 | 3.33 | 1.74 ± 0.77 | 1.00 | 2.00 | 2.83 | 3.83 | 4.83 | 2.91 ± 1.13 |
| - Women aged 18-40 yr | 1.00 | 1.17 | 1.67 | 2.33 | 3.33 | 1.85 ± 0.79 | 1.17 | 2.17 | 3.17 | 4.00 | 4.83 | 3.08 ± 1.11 |
| - Women aged 41-65 yr | 1.00 | 1.17 | 1.50 | 2.17 | 3.64 | 1.76 ± 0.80 | 1.00 | 2.00 | 2.83 | 3.83 | 4.83 | 2.86 ± 1.17 |
| - Men aged 18-40 yr | 1.00 | 1.17 | 1.33 | 1.83 | 3.17 | 1.57 ± 0.65 | 1.00 | 1.83 | 2.67 | 3.50 | 4.67 | 2.68 ± 1.07 |
| - Men aged 41-65 yr | 1.00 | 1.17 | 1.50 | 2.00 | 3.50 | 1.70 ± 0.76 | 1.00 | 1.83 | 2.67 | 3.67 | 4.83 | 2.81 ± 1.13 |
| ***Self-Harm*** | | | | | | | | | | | | |
| All participants | 1.00 | 1.00 | 1.00 | 1.00 | 1.50 | 1.07 ± 0.27 | 1.00 | 1.00 | 1.33 | 2.33 | 3.67 | 1.76 ± 0.96 |
| - Women aged 18-40 yr | 1.00 | 1.00 | 1.00 | 1.00 | 1.50 | 1.07 ± 0.27 | 1.00 | 1.00 | 1.33 | 2.33 | 4.00 | 1.78 ± 1.01 |
| - Women aged 41-65 yr | 1.00 | 1.00 | 1.00 | 1.00 | 1.67 | 1.09 ± 0.31 | 1.00 | 1.00 | 1.17 | 2.17 | 3.67 | 1.69 ± 0.92 |
| - Men aged 18-40 yr | 1.00 | 1.00 | 1.00 | 1.00 | 1.33 | 1.06 ± 0.26 | 1.00 | 1.00 | 1.50 | 2.33 | 3.67 | 1.79 ± 0.92 |
| - Men aged 41-65 yr | 1.00 | 1.00 | 1.00 | 1.00 | 1.50 | 1.05 ± 0.23 | 1.00 | 1.00 | 1.33 | 2.33 | 3.67 | 1.76 ± 0.93 |

ROM: Routine outcome monitoring
To calculate sum scores for the DAPP-SF subscales, multiply the mean scores by the number of items per subscale.
